# Reward sensitivity differs depending on global self-esteem in value-based decision-making

**DOI:** 10.1038/s41598-020-78635-1

**Published:** 2020-12-09

**Authors:** Aya Ogasawara, Yoshiyuki Ohmura, Yasuo Kuniyoshi

**Affiliations:** Intelligent Systems and Informatics Laboratory, Mechano-Informatics Department of Graduate School of Information Science and Technology, The University of Tokyo, Eng. Bldg.2, 7-3-1, Hongo, Bunkyo-ku, Tokyo, 113-8656 Japan

**Keywords:** Psychology, Human behaviour

## Abstract

Global self-esteem is a component of individual personality that impacts decision-making. Many studies have discussed the different preferences for decision-making in response to threats to a person’s self-confidence, depending on global self-esteem. However, studies about global self-esteem and non-social decision-making have indicated that decisions differ due to reward sensitivity. Here, reward sensitivity refers to the extent to which rewards change decisions. We hypothesized that individuals with lower global self-esteem have lower reward sensitivity and investigated the relationship between self-esteem and reward sensitivity using a computational model. We first examined the effect of expected value and maximum value in learning under uncertainties because some studies have shown the possibility of saliency (e.g. maximum value) and relative value (e.g. expected value) affecting decisions, respectively. In our learning task, expected value affected decisions, but there was no significant effect of maximum value. Therefore, we modelled participants’ choices under the condition of different expected value without considering maximum value. We used the Q-learning model, which is one of the traditional computational models in explaining experiential learning decisions. Global self-esteem correlated positively with reward sensitivity. Our results suggest that individual reward sensitivity affects decision-making depending on one’s global self-esteem.

## Introduction

Global self-esteem is a component of individual personality that has an impact on decision-making. Global self-esteem is defined as a general attitude of individuals regarding their own worth^1^. Low global self-esteem affects depression significantly^2^, and is related to risky behavior^3^; that is, global self-esteem is an influential predictor of mental and physical health.

Decision-making has social and non-social aspects. Many studies about the impact of global self-esteem on decision-making have focused on social aspects based on sociometer theory^4^. Individuals with a high global self-esteem tended to evaluate themselves more favorably than their partners’ rating^5,6^. On the other hand, individuals with lower global self-esteem believed that they received less positive feedback from peers indicating that they were liked or disliked^7^. Moreover, people with lower global self-esteem/higher anxiety showed greater self-other differences; namely, they preferred more risk-averse choices for personal decisions than that for others’ decisions^8,9^. In short, people with low global self-esteem tend to underestimate their own value, in response to feedback in social situations, compared to people with high global self-esteem.

However, there is also the possibility that individuals with lower global self-esteem underestimate reward and value equally, in both social and non-social situations. The relationship between global self-esteem and Behavioral Activation System (BAS) is notable. BAS is a model of appetitive motivation causing movement towards goals or rewards, assessed by the BAS scale^10^ which has 13 items (e.g., “When I see an opportunity for something I like, I get excited right away”). Some studies show that global self-esteem is positively related to BAS in self-report questionnaires^11,12^. These studies indicate that individuals with different global self-esteem have different responses to rewards. If people with lower self-esteem underestimate a received reward, a behavioral change by the reward has to be low even in actual decisions. The extent to which reward changes decisions was defined as reward sensitivity in our study. To our knowledge, there is no study to measure the relationship between global self-esteem and reward sensitivity behaviorally. In order to better understand the impact of global self-esteem on decision-making, it is necessary to investigate whether global self-esteem and reward sensitivity are correlated in terms of actual decision behaviors.

In general, computational models have been used to investigate decision-making^13,14^. One of the traditional computational models is the Q-learning model^15^, which has focuses on expected value and is quite successful in predicting choice behavior. In the learning paradigm, expected value and salience value might contribute to decision biases. Humans can learn about different payoffs through experience^16,17^. In addition, salience value affects decision biases and preference reversal under certain conditions^18^. Therefore, we should discuss global self-esteem and reward sensitivity by considering the impact of expected value and salience value. We adopted the maximum value as the salience value because maximum value, or large variance, was used for saliency in a previous study^18^.

In our study, we first examined the effects of expected value and salience value on learning under uncertainties, and then investigated the relationship between global self-esteem and reward sensitivity under the condition in which learning was affected, by using the Q-learning model^15^.

## Materials and methods

### Participants

We calculated the required sample size using G*Power^19,20^ and we needed 34 participants for an alpha of 0.05, a power of 0.80, and an effect size of 0.4. The effect size was defined based on the results of previous studies (e.g. the correlation coefficient between self-esteem and BAS with social desirability controlled was 0.54^12^, that between self-esteem and learning goal orientation was 0.4^21^). Therefore, we recruited thirty-seven participants (17 women and 20 men) with a mean age of 21.57 years (standard deviation, SD = 1.83) and all of them completed the task. All participants were right-handed, native Japanese speakers. They were all mentally healthy as determined through self-report.

This study was approved by the research ethics committee of University of Tokyo. The experiments were carried out in accordance with relevant guidelines and regulations based on the Declaration of Helsinki (1964). All participants provided written informed consent. After the experiment, they received monetary compensation for their time.

### Procedure

Participants answered questionnaires by the day of the experiment. The questionnaires included several scales, but we focused on the Rosenberg global self-esteem Scale (RSES)^1^. RSES is a questionnaire that assesses a person’s overall evaluation of his or her self-worth and is widely used to measure global self-esteem. It consists of 10 items, such as ‘On the whole, I am satisfied with myself’. We used the Japanese version^22^ and all responses were made on 5-point Likert-type scale (1: strongly disagree, 2: disagree a little, 3: neither agree nor disagree, 4: agree a little, and 5: strongly agree).

On the day of the experiment, participants were instructed for a task and engaged in a short training task to understand the flow of the behavioural task. After that, they were given the task whose duration was about one hour.

### Experimental task

Participants performed the behavioral task in which they chose from among different visual stimuli displayed on a computer monitor to get rewards. Participants were instructed to choose one of the stimuli to increase the total reward as much as possible. While participants were told that payoffs depended only on the visual stimuli, not its location or their history of choices, they were never informed about the payoffs associated with the different stimuli and had to estimate them from experience.

In order to investigate the contributions of the expected value and the maximum value, we prepared three conditions; each stimulus had the same expected value and a different maximum value (condition 1; C1), each stimulus had the same expected value and the same maximum value (condition 2; C2), each stimulus had a different expected value and the same maximum value (condition 3; C3). The influence of the maximum value was investigated by comparing C1 and C2, and the influence of the expected value was investigated by comparing C2 and C3. The order of conditions was randomized for each participant.

Under each condition, there were four visual stimuli associated with different discrete probability distributions. Visual stimuli were similar to those used in a previous study^17^, and were different depending on conditions. Payoffs of each condition are listed in Table 1. In ascending order of the probability of values lower than expected values, we labeled them ‘sure stimulus’, ‘low uncertain stimulus’, ‘medium uncertain stimulus’, and ‘high uncertain stimulus’. Therefore, there were six combinations under each condition. Each combination was displayed 20 times randomly. Stimuli were displayed on both the left and right sides of the screen, and sides of stimuli were randomly switched. In other words, each condition consisted of 120 trials.

**Table 1:** Payoffs in each condition.

Condition	Payoff	Expected value	Maximum value
C1	60 (1) or 30 (0)	60	60
	70 (3/4) or 30 (1/4)	60	70
	90 (1/2) or 30 (1/2)	60	90
	150 (1/4) or 30 (3/4)	60	150
C2	90 (0) or 60 (1) or 30 (0)	60	90
	90 (1/8) or 60 (3/4) or 30 (1/8)	60	90
	90 (1/4) or 60 (1/2) or 30 (1/4)	60	90
	90 (3/8) or 60 (1/4) or 30 (3/8)	60	90
C3	90 (1) or 30 (0)	90	90
	90 (3/4) or 30 (1/4)	75	90
	90 (1/2) or 30 (1/2)	60	90
	90 (1/4) or 30 (3/4)	45	90

Numbers in parentheses signifying probability of each payoff.

**Figure 1:**
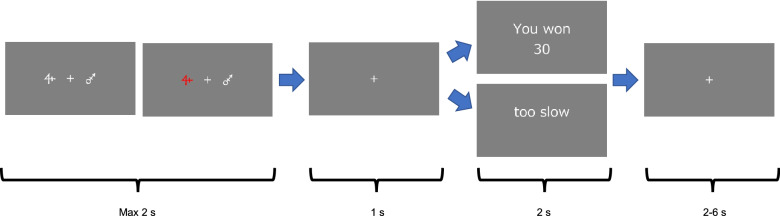
Schematic representation of the experiment. Participants chose one of the visual stimuli within 2 s. The colour of the selected stimuli turned red. Then the fixation cross was presented for 1 s, and feedback was displayed for 2 s. The next trial began after a variable interval of 2–6 s.

The schematic representation of the experiment is provided in Fig. 1. Participants could take breaks between conditions and start the next condition by pressing the space key. In each trial, participants had to select one of them by pressing the key within 2 s. The spacebar, “j” and “f” keys were labelled “start”, “right” and “left”, respectively. Participants could select the right stimuli by pressing the “j” key and the left stimuli by pressing the “f” key. The color of the selected stimuli changed after either key was pressed. Then the fixation cross was presented for 1 s, and the payoff associated with the chosen stimulus was displayed for 2 s. If participants did not select a stimulus, “too slow” was displayed. After a variable inter-trial interval of 2–6 s, the next trial began. When the session was over, the total rewards in the session was displayed for 5 s.

The short training task had different payoffs and visual stimuli from the behavioral task. Participants played 6 trials to understand the flow of the behavioral task and keys’ positions.

### Behavior analysis

We analyzed reward sensitivity of value-based decision-making in two steps. First, in order to investigate whether maximum values and expected values affected decisions in the learning paradigm, we compared ratios of choices of more uncertain stimuli between C1 and C2, and between C2 and C3, respectively. C1 and C2 had the same expected values and different maximum values, and C2 and C3 had the same maximum values and different expected values. We performed two-sample Kolmogorov–Smirnov test to examine whether decisions were from different continuous distributions. The threshold was set at p < 0.05.

Second, we investigated whether global self-esteem was correlated with reward sensitivity. We used Q-learning model^15^ and modelled the condition that had significant effect on decisions at the first step. This model offers a general framework for trial-and-error learning of value-based decision-making. On trial *t*, participants had an expectation ($Q_i(t)$) of the average reward they might gain from visual stimulus *i*. After every choice, a prediction error $\delta (t)=r(t)-Q_C(t)$ was computed using the expectation $Q_C(t)$ of the chosen stimulus *C*, where *r*(*t*) was the reward at trial *t*. Prediction error $\delta$ was used to update $Q_C(t)$, the expectation of the chosen stimulus, as follows:

1 $$\begin{aligned} Q_C(t+1)=Q_C(t)+\alpha \cdot \delta \end{aligned}$$

with $\alpha$ being the learning rate referring to the weight given to presented reward on a given trial. In other words, the learning rate represents how much the current trial’s reward affects the next decision. Here, reward sensitivity refers to the extent to which the reward changes decisions. Therefore, we used the learning rate as reward sensitivity. If a participant did not select one of stimuli within 2 s, $Q_C(t)$ was not updated. Additionally, the probability ($P_i(t)$) of choosing stimulus *i* was derived using a softmax action selection function:

2 $$\begin{aligned} P_i(t)=\frac{1}{1+exp(-Q_i(t)-Q_j(t))} \end{aligned}$$

where *i* and *j* were displayed stimuli ($i\ne j$) on trial *t*.

We fitted each participant’s parameter $\alpha$ because we were interested in inter-participant differences of reward sensitivity. We performed a grid search to find the best parameter by minimizing the likelihood function:

3 $$\begin{aligned} L=\sum _{t=1}^{T} \ln P_C(t) \end{aligned}$$

where T denoted the total number of trials of the condition, which was 120 in our experiment. We varied reward sensitivity $\alpha$ within the range [0.00001 1] in increments of 0.00001. We calculated Spearman’s correlation between RSES score and reward sensitivity $\alpha$. The threshold was set at p < 0.05.

We used the bootstrap method^23^ to estimate statistics on a population. We resampled 37 participants with duplication for 1000 times and created an estimated distribution of each statistic value. We used percentile bootstrap confidence intervals (1 − *a*) to determine whether results were significant. The significant threshold was $a<$0.05. If Kolmogorov–Smirnov test statistics and the correlation coefficient are significant even with the resampling method, our results were sufficiently reliable.

## Results

### The effect of expected value and maximum value

**Figure 2:**
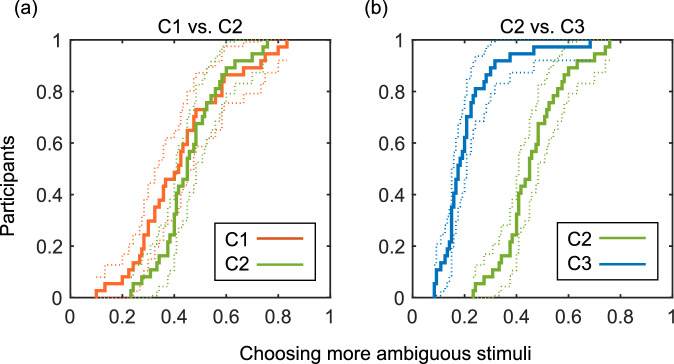
Decisions of each condition. Empirical cumulative distribution functions for ratios of choices of more ambiguous stimuli. The vertical axis represents the proportion of participants. (**a**) Decisions of C1 and C2. (**b**) Decisions of C2 and C3.

There was no significant difference in decisions between C1 and C2 (p $=$ 0.061) (Fig. 2a). On the other hand, C2 and C3 had significantly different continuous distributions of decisions (p < 0.001) (Fig. 2b). Therefore, in this learning paradigm, only expected value affected decisions between different uncertainties. These results were verified by the resampling analysis (C1 vs. C2: $a>$ 0.1, C2 vs. C3: $a<$ 0.001).

### Global self-esteem and reward sensitivity

**Figure 3:**
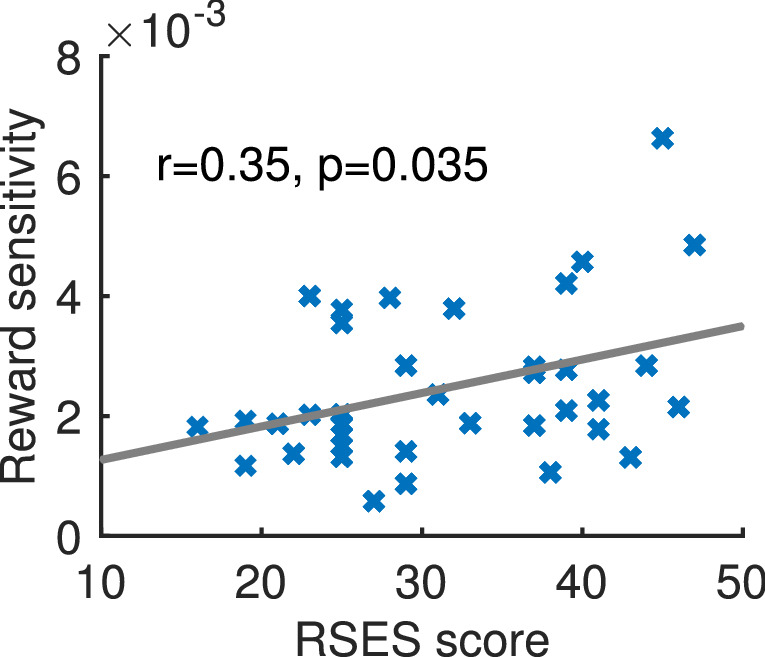
Reward sensitivity and RSES score (r $=$ 0.35, p $=$ 0.035).

The mean RSES score was 31.59 (SD = 8.70) and there was no significant gender difference (t = 0.37, p = 0.71). Although we assumed that the maximum values could affect value-based decision-making, only expected values affected decisions in our learning paradigm. Therefore, we modelled participants’ decisions under C3 that had different expected values without considering maximum value. Participants selected stimulus which had higher expected values significantly ($\hbox {t}=15.68$, $\hbox {p}<0.001$). The mean reward sensitivity was 0.00247 (SD = 0.00129). RSES score and reward sensitivity were significantly positively correlated (r $=$ 0.35, p $=$ 0.035) (Fig. 3). These results were verified by the resampling analysis ($a<$ 0.05).

## Discussion

We used a value-based decision-making task with controlled expected value and maximum value in order to investigate the relationship between global self-esteem and reward sensitivity. In this experiment, only expected value affected decisions. Therefore, we modelled decisions under the condition with different expected values using Q-learning model and calculated the correlation between global self-esteem and reward sensitivity. Based on the results, global self-esteem and reward sensitivity were significantly positively correlated.

Our results showed that only expected value affected value-based decision-making in our learning paradigm. It is rational to make decisions based on expected value. In fact, many studies have shown that people could choose a significantly higher expected reward than the chance level^17,24^. Contrarily, some studies showed that human decision-making was not fully explained only by expected value. The main reason why our results did not show the effect of maximum value might be that our experiment involved a learning paradigm. Uncertainty due to missing probability information is often called ambiguity. Tsetsos et al.^18^ showed the impact of saliency by letting participants choose one sequence after simultaneously viewing two or three rapidly varying sequences of numerical values. This is a one-shot decision-making. Moreover, Frisch and Baron^25^ insisted that ambiguity did not threaten the normative status of utility theory. Therefore, saliency might not affect repeated choices in the condition that has the same expected value and different maximum value. Similarly, saliency did not have the relationship with global self-esteem in our task (see Supplementary Information S1 for detailed explanation).

The most notable result was that RSES score correlated positively with reward sensitivity. This result supported the possibility suggested by previous studies about global self-esteem and self-report questionnaires^11,12,26^, that is, the different preferences for decisions depending on global self-esteem due to how to receive presented reward. Our finding indicated new possibilities in understanding of the role of reward sensitivity in decision-making related to global self-esteem. On the other hand, many studies showed that global self-esteem biased feedback in social contexts. Individuals with low global self-esteem believed that they received less positive feedback^7^, and those with high global self-esteem estimated themselves to be more favourable than their partners’ rating^5,6^. In addition, global self-esteem reflects an accumulation of past appraisals from others^27,28^, and fluctuations in self-esteem depended on prediction errors between expected and received social feedback^29^. The further research considering these insights and our results together may lead better understanding the role of self-esteem for decision-making.

There were several limitations in the present study. First, there may be other possible models to explain reward sensitivity. For example, the impact of global self-esteem on decisions under a risk was different between the gain frame and loss frame^9^. In general, reward sensitivity and risk sensitivity were different when people learned outcomes of their actions through trial and error^30^. In addition, some studies have claimed that learning rate and reward sensitivity should be separated^31^. Although this model is unsuitable for our behavioral task (See Supplementary Information S1 for detailed explanation), it is true that there are other possible formulations to explain the relationship between global self-esteem and decision-making. In our study, we clarified the difference in reward sensitivity with the simplest way, that is, the extent to which reward changes decisions in the gain frame, so more detailed behavior-based research of reward sensitivity is necessary. Second, while our experiment involved numerical feedback, global self-esteem is also closely related to social feedback from others for example, low global self-esteem enhances social pain^32^.

Although there were several limitations, our results are important to understand the impact of global self-esteem on value-based decision-making. To the best of our knowledge, because there has been no research to model the relationship between global self-esteem and reward sensitivity using a computational model, prior to this. In particular, our results suggested that inter-individual difference in global self-esteem might contribute to reward sensitivity.

## Supplementary information

Supplementary Information

## Data Availability

The data sets generated during the current study are available from the corresponding author upon reasonable request.
