# Supplementary material for: Reward sensitivity differs depending on global self-esteem in value-based decision-making
Source: Sci Rep. 2020 Dec 9;10:21525. doi: 10.1038/s41598-020-78635-1 (PMC7725803; doi:10.1038/s41598-020-78635-1)
Supplement: Supplementary file 1 — Supplementary Information [file 41598_2020_78635_MOESM1_ESM.pdf]

Reward sensitivity differs depending on global self-esteem in value-based decision-making

Aya Ogasawara, Yoshiyuki Ohmura, Yasuo Kuniyoshi

Supplementary Information

In this section, we discuss the relationship between reward sensitivity and learning rate. In our study, we have defined reward sensitivity as the extent to which reward changes decisions. One way to evaluate it objectively is to use behavioral indicators, and some studies attempt to separate reward sensitivity from the learning rate. However, these models are still not established. There are several reasons.

First, some studies have suggested that numeral rewards used in our experiment may be different from hedonic stimuli which activate liking system[1] although one reason for separating reward sensitivity and the learning rate is that 'liking' is dissociable from 'wanting' and learning[2]. Second, behavioral change itself depends on reward sensitivity and in the formula, reward sensitivity and learning rate are proportional. In our main article, we used the traditional Q-learning model[3]. The formulation was as follows. On trial t, participants had an expectation ($Q_i(t)$) of the average reward they might gain from visual stimulus i. After every choice, a prediction error $\delta(t) = r(t) - Q_C(t)$ was computed using the expectation $Q_C(t)$ of the chosen stimulus C, where $r(t)$ was the reward at trial t. $Q_C(t)$ was updated in two ways. One is a traditional Q learning model[3]. Prediction error δ was used to update $Q_C(t)$, the expectation of the chosen stimulus, as follows:

$$Q_C(t+1) = Q_C(t) + \alpha \cdot \delta \tag{1}$$

with α being the learning rate referring to the weight given to presented reward on a given trial. On the other hand, a slightly different formula dividing learning rate and reward sensitivity were often used in mental disorders[2]. In this model, a prediction error was $\delta'(t) = \rho \cdot r(t) - Q_C(t)$. $Q_C(t)$ was updated as follows:

$$Q_C(t+1) = Q_C(t) + \alpha' \cdot \delta' \tag{2}$$

Here, α' was the learning rate and ρ was reward sensitivity. As you can see, where the effect of the presented reward on decisions $\alpha' \cdot \rho$ was the same meaning as the learning rate α. In addition, the biggest problem when using a model that separates reward sensitivity and learning rate is that our simple behavioral task becomes an ill-posed problem because only the differences in $Q_i(t)$ of the presented choices are used to make decisions, indicating that absolute value of $Q_i(t)$ is arbitrarily determined by model fitting.

In order to validate the model with the learning rate as reward sensitivity, we compared those models using AIC[4] under each condition. In both models, if a participant did not select one of the stimuli within 2 s, $Q_C(t)$ was not updated. Additionally, the probability ($P_i(t)$) of choosing stimulus i was derived using a softmax action selection function:

$$P_i(t) = \frac{1}{1 + exp(-Q_i(t) - Q_j(t))} \tag{3}$$

where i and j were displayed stimuli ($i \neq j$) on trial t. We fitted each participant's parameter α, α', and ρ because we were interested in inter-participant differences of reward sensitivity. We performed a grid search to find the best parameter by minimizing the likelihood function:

$$L = \sum_{t=1}^{T} \ln P_C(t) \tag{4}$$

where T denoted the total number of trials of the condition, which was 120 in our experiment. We varied α and α' within the range [0.00001 0.001] in increments of 0.00001. This range was decided based on the range of α obtained in the main article. On the other hand, we decided the range of ρ within the range [0.1 2]. We performed the grid search of ρ in increments of 0.1.

As a result, the total AICs for all participants were lower in equation (1) than in equation (2) for all conditions (Table 1). In other words, the model of equation (1) was better in our experiment.

For these reasons, it is unreliable for our study to separate reward sensitivity and learning rate and we believe that it should be withheld at this stage to use a formulation that separates reward sensitivity from learning rate when behaviorally assessing reward sensitivity.

In addition, there were no significant correlations between RSES score and learning rate in equation (1) of C1 and C2 ($p>0.1$). Therefore, we did not find the relationship differences in global self-esteem and preferences for maximum value.

Condition	AIC in equation (1)	AIC in equation (2)
C1	4592	4643
C2	4964	5020
C3	3702	3757

Table 1. The total AICs in each condition.

References

1. Buchel, C., Miedl, S. & Sprenger, C. Hedonic processing in humans is mediated by an opioidergic mechanism in a mesocorticolimbic system. *ELife* **7**, e39648 (2018).

2. Chen, C., Takahashi, T., Nakagawa, S., Inoue, T. & Kusumi, I. Reinforcement learning in depression: a review of computational research. *Neurosci. & Biobehav. Rev.* **55**, 247–267 (2015).

3. Sutton, R. S., Barto, A. G. *et al. Introduction to reinforcement learning*, vol. 135 (MIT press Cambridge, 1998).

4. Akaike, H. Information theory and an extension of the maximum likelihood principle. In *Selected papers of hirotugu akaike*, 199–213 (Springer, 1998).
